# An insight on established retinal injury mechanisms and prevalent retinal stem cell activation pathways in vertebrate models

**DOI:** 10.1002/ame2.12177

**Published:** 2021-07-09

**Authors:** Rinchen Doma Sherpa, Subhra Prakash Hui

**Affiliations:** ^1^ S. N. Pradhan Centre for Neurosciences University of Calcutta Kolkata India

**Keywords:** animal models, retina injury, retina regeneration, retinal stem cells, zebrafish

## Abstract

Implementing different tools and injury mechanisms in multiple animal models of retina regeneration, researchers have discovered the existence of retinal stem/progenitor cells. Although they appear to be distributed uniformly across the vertebrate lineage, the reparative potential of the retina is mainly restricted to lower vertebrates. Regenerative repair post‐injury requires the creation of a proliferative niche, vital for proper stem cell activation, propagation, and lineage differentiation. This seems to be lacking in mammals. Hence, in this review, we first discuss the many forms of retinal injuries that have been generated using animal models. Next, we discuss how they are utilized to stimulate regeneration and mimic eye disease pathologies. The key to driving stem cell activation in mammals relies on the information we can gather from these models. Lastly, we present a brief update about the genes, growth factors, and signaling pathways that have been brought to light using these models.

## Graphical abstract

Retinal stem cells (RSC) has been found to exist throughout the vertebrate lineage but the ability to repair injured retina is restricted to lower vertebrate groups. Experiments on regeneration competent animal models pave the way for understanding the underlying mechanisms behind successful retina regeneration which in turn serve as a potential future therapy in humans.

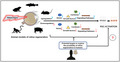

## 1 INTRODUCTION

The brain processes visual information when light energy transduces into neural activity in the retina. The close‐knit components of the central nervous system (CNS), the brain, and its extension retina are thus the critical players in visual perception, thereby aiding in daily activities. While the brain remains well protected inside the skull, the eyes are quite susceptible to physical injuries and chemical accidents.^1^ Furthermore, one's genetic makeup and increasing age also invite multiple numbers of eye diseases such as retinitis pigmentosa (RP), age‐related macular degeneration (AMD), glaucoma, etc All this has contributed to the recent “World Reports on vision (2019),” which shows that a whopping 2.2 billion people globally fell victim to visual impairment in the past year.^2^ The discovery of the existence of adult retinal stem/progenitor cells among different vertebrate species^3^ and its high reparative activity in the case of lower vertebrates has presented us with a possibility to “self‐heal” the retina one day.^4^ Consequently, high regeneration competent animals, which include the amphibian newts and *Xenopus*, teleost zebrafish (*Danio rerio*), and chick are thus being explored^5^ to investigate different genetic and epigenetic features, signaling pathways, and factors^6^, ^7^ that regulate stem cell activation, thus gradually filling in the gaps of our knowledge of mammals, which appear to be the least competent among the group.^8^ With the hope of updating and giving researchers an idea about how these animal models have significantly shaped our understanding of the retinal regeneration process, in this review, we discuss the different developed injury mechanisms and then talk about underlying genes, growth factors and signaling pathways involved in retinal stem cell activation in different animal models.

## 2 MECHANISMS OF RETINAL INJURY

The injury mechanism of the retina has shown a wide range of variations ranging from whole‐cell retinal injury to exploiting each layer individually viz. photoreceptor,^9^, ^10^, ^11^ ganglionic layer,^12^ etc, using light, chemicals, transgenic lines, and genetic ablations. Various injury methods were applied with the following aims:

1. To determine where the regeneration capacity lies within the retina.
2. To study regenerative response during different modes of injury and be able to mediate whole‐cell as well as targeted injuries, thereby mimicking diverse retinal diseases.

### 2.1 Mechanical injury

Successful experiments with autoplastic eye implantation in salamander larvae and restoration of optic nerve severing in newt^13^ were among the first significant research that provided evidence for the regenerative capabilities of the eye. Following these experiments, numerous amphibian and teleost fish models have been exploited using the mechanical mode of injury.^14^, ^15^

**FIGURE 1:**
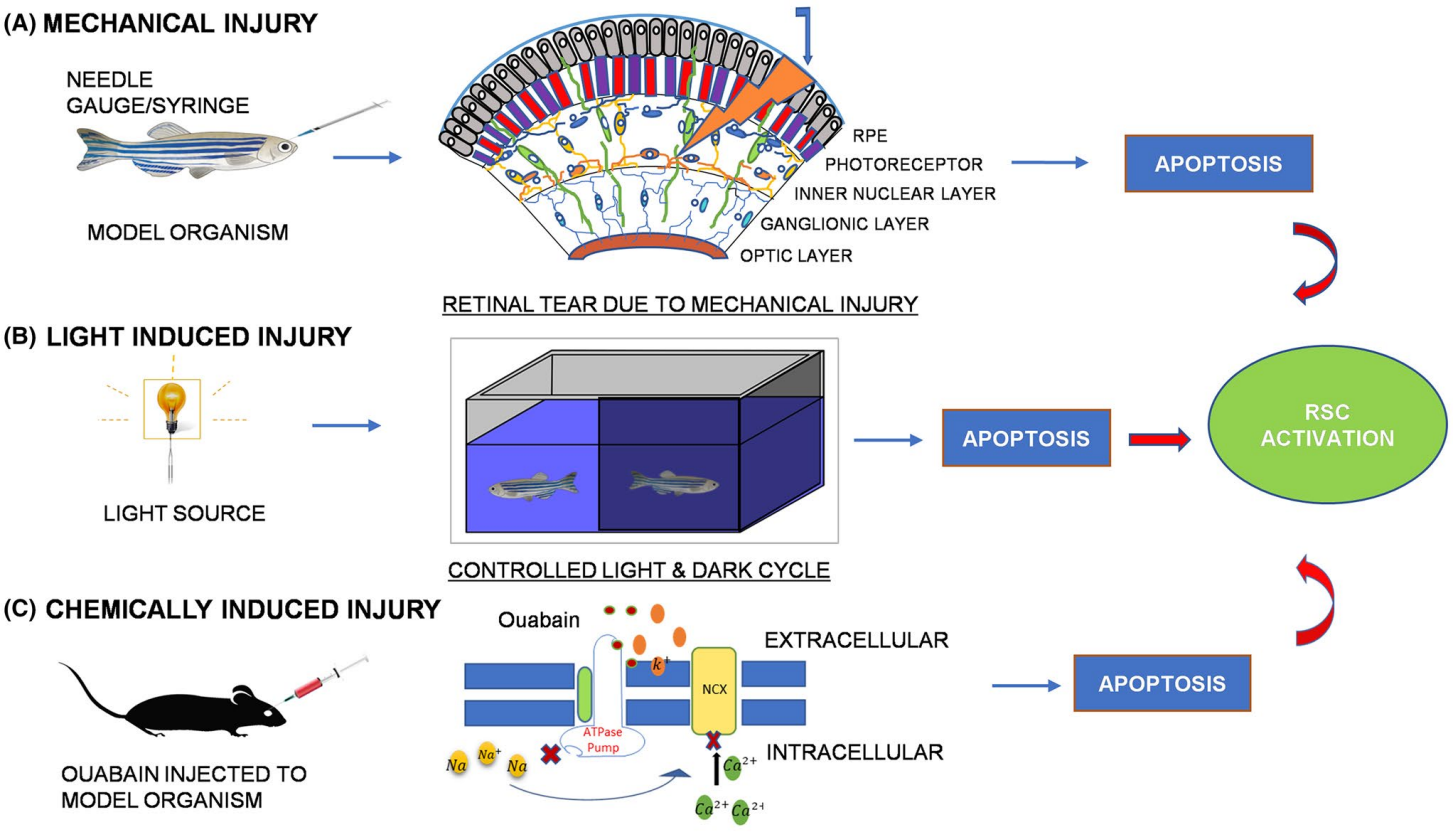
An illustrative representation of mechanical, light, and chemical induced injury models of the retina. A, In mechanical poke injury, a needle gauge/ syringe is used to poke a hole in the retina that causes uniform damage to all layers. B, In light‐induced injury, disruption of the light/dark cycle is done by exposing model organisms to high‐intensity light for varied periods. C, In chemically induced injuries such as ouabain toxicity, tissues of model organisms injected with ouabain show cellular apoptosis due to ouabain‐mediated blocking of sodium‐potassium ATPase pump, which causes an increase in intracellular sodium ion concentration that in turn inhibits the function of the sodium‐calcium exchanger (NCX)

Mechanical injury of the retina is achieved through surgical procedures such as incisions, poke as exemplified in Figure 1A, or removal of a small part of the retina. The past two decades have witnessed various methods of retinal injuries such as transscleral injuries, poke injuries,^16^, ^17^, ^18^, ^19^ and retinal detachments^20^, ^21^ as a result of the experiments being performed on different animal models. Transscleral injury involves using a microknife to excise a small flap of the retina, where local excision of the retina with all the layers is done and used for the study of neuroretina regeneration.^22^ In poke or stab injury, eyeballs of the animal model are tilted with forceps and stabbed on the edges with a syringe, thus inflicting damage to all layers. Retinal incisions followed by subretinal injections of saline and hyaluronic acid help create retinal detachment, that is, separation of the neural retina from the underlying retinal pigment epithelium (RPE).^20^, ^21^ This injury method helps study changes in photoreceptor outer segment apoptosis and regeneration.^21^, ^23^ The use of mechanical injury for retina regeneration is one of the oldest yet most feasible injury mechanisms. Moreover, it is the best option for someone aiming to study whole‐cell retinal injury because it achieves uniform damage to the retina.^16^, ^17^, ^24^

### 2.2 Light‐induced injury

Light is the environmental stimulus that is necessary for vision. However, constant long‐term exposures or high‐intensity light can be very damaging to the recipient's photoreceptor layer.^25^ In the electromagnetic spectrum, the range of light between 400 and 1400 nm is the “retinal hazard region” as it is the range that is allowed to pass to the retina.^26^ Although this range consists of only visible and short wavelength infrared light, visual complications may arise depending on the intensity of light and exposure time. Owing to its high exposure rate in humans, the blue light component (415‐495 nm) has also been experimented with in animal models and has been shown to inflict damage to the photoreceptors and the retinal pigment epithelium layer as well.^27^, ^28^, ^29^

Light‐induced injury mechanism usually follows a disruption of the standard 10 hours light‐14 hours dark cycle^30^ to a long dark cycle followed by exposure to high‐intensity visible light^9^, ^31^ or ultraviolet (UV) light.^32^ Temporal variations for light‐induced injury include long or short durations of exposure^27^ that are repeated once or several times^10^, ^33^ depending on the experimental setup as exemplified by Figure 1B. The light source may vary from tungsten halogen lamps,^10^, ^31^, ^32^ to metal halide lamps,^9^ and fiber optics,^34^ with light intensities at the water interface being as great as 100 000 lux.^32^

The 3 modes of light‐induced injury include:

#### 2.2.1 Photomechanical injury

Laser‐based irradiation of retinal pigment epithelium (RPE) has the ability to cause 2 distinct modes of cell damage depending on the time of exposure. These are thermal denaturation (exposure duration more than 10 µs) and intracellular cavitation (exposure duration below 10 µs).^35^ For exposure duration of up to 10 µs, which falls in the order of the thermal relaxation period of RPE, melanosomes show a very high increase in temperature ranging up to 150°C.^36^, ^37^ Thus, cytoplasm contact with these melanosomes undergoes rapid vaporization, thereby creating microcavitation bubbles.^36^ Rapid expansion and dissolution of these bubbles cause mechanical damage and induce apoptosis in the underlying RPE cells via disruption of lysozymes.^36^

#### 2.2.2 Photochemical injury

Post light exposure, dissipated energy from excited chromophores such as lipofuscin and flavoprotein may lead to the production of reactive oxygen species (ROS).^26^, ^38^ These chemicals are highly damaging to all cell types, and in the retina, they may initiate apoptosis of the light recipient photoreceptor layer.^26^

#### 2.2.3 Photothermal injury

Light in the form of photons is capable of increasing the mean kinetic energy of the recipient molecules. When this energy dissipates, molecular collisions lead to an increase in these molecules’ temperature, thereby causing thermal damage to the cells involved.^26^, ^38^ This injury mode is observed in laser light photocoagulation and optical coherence tomography (OCT)‐guided laser injuries and has been experimented with in many animal models.^39^, ^40^

### 2.3 Chemical injury

Although both mechanical and light injury can be used to target different retinal layers, mechanical injury best portrays whole‐retina damage as, for light‐induced injury, photoreceptor‐specific damage can be well modeled. Chemical injury, on the other hand, by virtue of trial‐and‐error quantification of doses can precisely damage any retinal layer and thus presents a chance for mimicking multiple eye pathologies depending on the targeted layer. Popular choices of chemicals include ouabain, 6‐hydroxydopamin (OHDA), hypoxia‐inducing chemicals, *N*‐methyl‐d‐aspartate (NMDA), nitroreductase/metronidazole (NTR/Mtz). Depending upon the targeted area and the animal model being used, these chemicals are quantified accordingly.

#### 2.3.1 Ouabain‐mediated chemical injury

The cardiac glycoside ouabain acts by causing the inhibition of Na^+^/K^+^ ATPase, hence acting as a metabolic poison by increasing intracellular Na^+^ ion concentration that inhibits the sodium‐potassium exchanger^41^ as shown in Figure 1C. Introduced by Maier and Wolberg in 1979,^42^ it can destroy the whole retina^43^ when used in high doses, and intravitreal injection of lower doses is efficient in targeting different individual layers such as the inner nuclear layers,^44^ amacrine layers,^12^ and photoreceptors.^11^, ^42^ The injury mechanism usually follows a microknife for incision followed by injection of ouabain in the intravitreal cavity.

#### 2.3.2 6‐OHDA mediated injury

Dopaminergic neurons are distributed throughout the retina and play an essential role in the growth and survival of retinal cells. 6‐OHDA is a neurotoxin that targets noradrenergic and dopaminergic neuron destruction.^45^, ^46^ The injury mechanism, similar to ouabain treatment, follows the making of scleral incisions using a microknife and then microsyringe‐mediated administration of the chemical. 6‐OHDA is quickly converted to its quinone form in solution, thereby generating free radicals. Hence, while using relatively higher doses of 6‐OHDA, sodium ascorbate is added to slow down the build‐up of these autoxidation products that may cause nonspecific damage.^46^

#### 2.3.3 Chemically induced hypoxia

Cobalt chloride (CoCl_2_) prevents iron inclusion in the heme, thereby decreasing hemoglobin that carries oxygen to different parts of the body.^47^ This causes hypoxia, leading to the production of hypoxia‐inducible factors (HIF). Furthermore, CoCl_2_ also inhibits the proteasome‐mediated degradation of HIF, thereby causing hypoxic injury.^48^ HIF, in turn, stimulates the production of vascular endothelial growth factors that causes neovascularization and aberrant angiogenesis.^49^ CoCl_2_‐mediated hypoxic injury involves intravitreal administration and has been used to target different retinal layers such as the photoreceptors^11^ and ganglionic layers.^50^

#### 2.3.4 NMDA receptor‐mediated injury

High doses of NMDA are known to cause NMDA receptor‐mediated influx of cations in massive amounts that lead to overexcitation of synapses, causing neuronal death.^51^ Based on this principle, NMDA has been used to injure animal models in different retinal layers such as the rods of the photoreceptor layer or retinal ganglionic layer as well as other layers.^52^, ^53^

#### 2.3.5 NTR/Mtz‐mediated cell ablation

This injury lies in the ability of the *Escherichia coli* bacteria's nitroreductase (NTR) enzyme to reduce prodrug metronidazole (Mtz) into a cytotoxic DNA cross‐linking agent, the expression of which causes cellular apoptosis. The technique is used to ablate various types of retinal cells such as the ultraviolet cones,^54^ blue cones,^55^ rods,^56^, ^57^ and bipolar cells.^58^ *Tg(zop:nfsB)*EGFP^nt20^ and *Tg(zop:nfsB)* EGFP^nt19^ are examples of 2 such transgenic lines that express the NTR promoter in the rod cells.^56^, ^59^

Chemical injury in the case of CoCl_2_ can be best correlated with retinopathy of prematurity in which the retina in the infants is hypoxic because the central retinal vessels cannot reach the periphery, leading to infantile blindness,^60^ whereas ouabain, 6‐ODHA, NMDA, and NTR/Mtz induced cell ablations create a pathological environment that induces the regeneration process.^12^, ^43^, ^55^, ^59^

### 2.4 Genetic models of retina diseases

**FIGURE 2:**
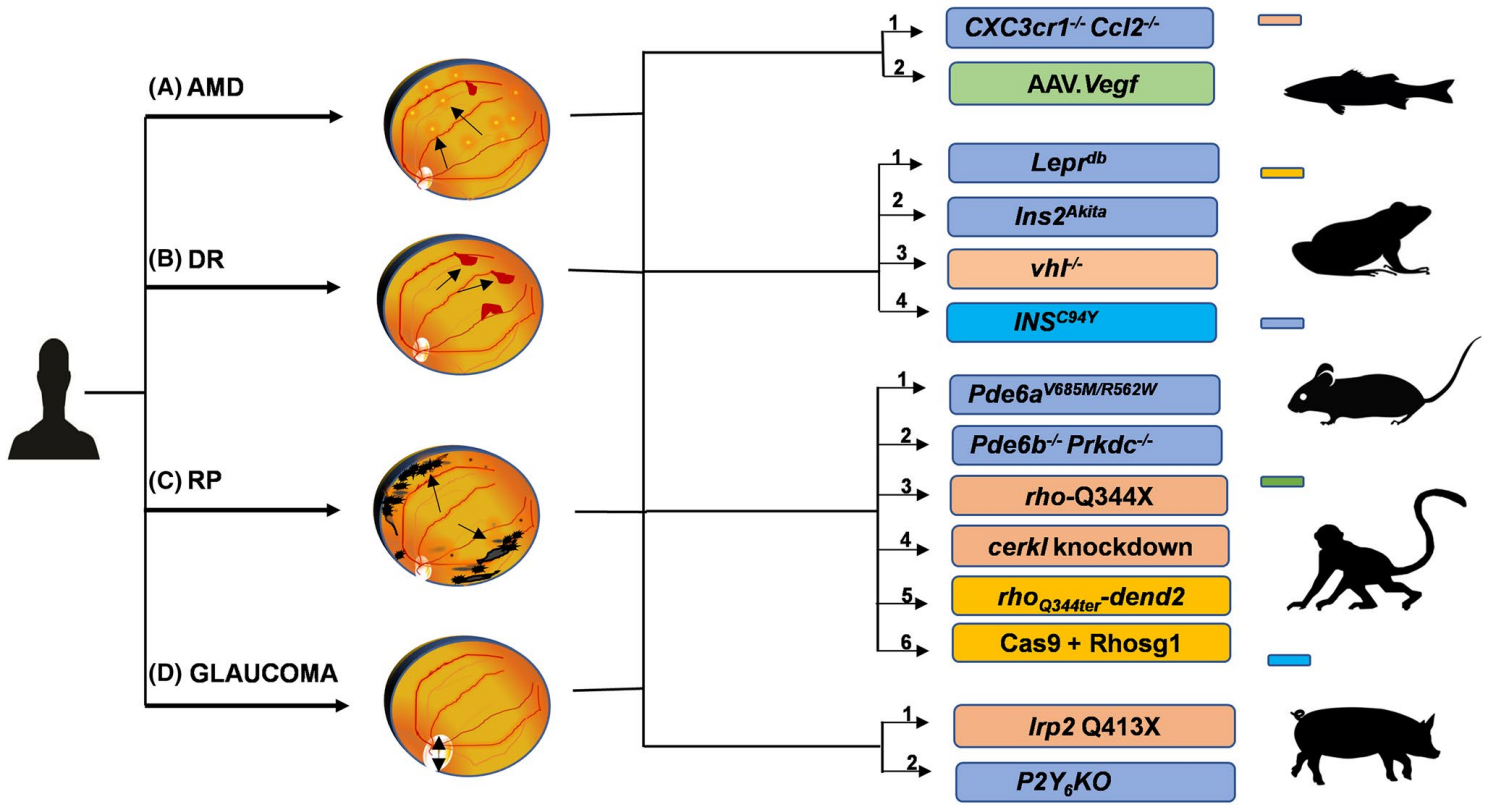
Genetic models of retinal diseases. A, An ophthalmoscopic view of the eye during AMD as identified by yellow extracellular membrane accumulation/drusen deposits: [1] CXCR1 is a CX3CR1/fractalkine chemokine receptor and has shown low transcript levels and SNPs during AMD; CCL2 is CC chemokine thought to play an immunoregulatory role in AMD *CXC3cr1* ^−/−^ *Ccl2* ^−/−^ are thus homozygous mice created to model AMD. [2] The nonhuman primate Rhesus monkeys have been used to model AMD, DR, etc via AAV *Vegf* expression which controls neovascularization. (CXCR1: C‐X‐C motif chemokine receptor; CCL2: C‐C motif ligand 2 chemokine; AAV‐Vegf: adeno‐associated viral vector mediated vascular endothelial growth factor expression). B, An ophthalmoscopic view of the eye during DR as identified by neovascularization and leaky blood vessels: [1] Leptin receptors are essential regulators of obesity; hence, mouse models of chronic hyperglycemia have been modeled in *Lepr^db^* mouse mutant for leptin receptor. [2] *Ins2^Akita^* with missense mutation in insulin 2 gene is another mouse DR model. [3] Von Hippel Lindau protein regulates Hif levels that can induce angiogenesis, *vhl* ^−/−^ biallelically inactivated zebrafish thus shows DR phenotype. [4] Large animal model of DR is the *INS* ^C94Y^ transgenic pig created by mutating the insulin gene. (Lepr: leptin receptor; Ins/INS: insulin; vhl: von Hippel Lindau). C, An ophthalmoscopic view of the eye during RP identified by the presence of intraretinal melanin deposits/bone spicule pigments, a hallmark of RP: [1] Mutations in the *Pde6a* and *Pde6b* gene are known to cause RP. The compound heterozygote RP mice model *Pde6a* ^V685M/R562W^ is an exact homolog of human RP. [2] *Pde6b* functions in the photo cascade transduction, and Prkdc encodes for catalytic subunit of DNA‐dependent protein kinase DNA‐PK. The *Pde6b^−/−^ Prkdc* ^−/−^ is an immunocompromised mice model of RP that lacks T, B, and NKT cells. [3] This transgenic zebrafish model expresses a mutated human rhodopsin gene construct Rho‐Q334X to model RP. [4] Single mutation in some genes such as *cerkl* has been shown to cause autosomal dominant RP. Thus, morpholino knockdown of *cerkl* has been used to mimic RP in zebrafish. [5] Rhodopsin mislocalization is one of the observed features of RP based upon this human class 1 rhodopsin mutation *Q344ter* has been fused to *dendra 2* fluorescent protein to mimic RP in frog. [6] CRISPR‐Cas9 has also been used in frogs to create point mutation in rhodopsin gene and model RP phenotype in the animal model. (Pde6a/b: phosphodiesterase 6; Prkdc: protein kinase DNA‐activated catalytic subunit; Rho: rhodopsin; cerkl: ceramide kinase like). D, An ophthalmoscopic view of the eye during glaucoma characterized by a relatively large optic cup resulting from loss of optic nerve due to high intraocular pressure. [1] The low‐density receptor‐related lipoprotein mutation at Q413X shows glaucoma‐like phenotype in zebrafish. [2] Purinergic receptor *P2Y*_6_ is important in regulating the intraocular pressure, and thus its knockout has been used to model the disease in mice. (P2Y: purinergic receptor)

With the help of genetic screening tools, scientists have collected numerous data and identified specific genes that are involved in certain retinal diseases.^61^ Inherited retinal diseases such as achromatopsia, RP, Leber's congenital amaurosis (LCA), AMD, etc comprise an extensive collection of heterogeneous mutations involving almost 250 genes.^62^ Besides, eye diseases such as diabetic retinopathy (DR) and glaucoma also contribute highly to eye diseases worldwide.^63^ Experiments for treatment of such diseases would require disease modeling, which is made possible by creation of knockdown, knock‐in, or insertion of mutated variants in the model organism. The science of transgenics, in which foreign genes can be inserted and expressed successfully in some other organism, and other genome editing techniques such as morpholino‐based gene silencing, transcription‐activator like effector nucleases (TALENs), and clustered regularly interspaced short palindromic repeats (CRISPR‐Cas) enzyme system make possible the imitation of diseases in a model organism.^64^, ^65^, ^66^, ^67^, ^68^, ^69^ The creation of such genetic models of a disease hence provides a platform for novel therapeutics.^63^ Here we have talked about a few genetic models of RP and DR; furthermore, a few examples from AMD^70^, ^71^ and glaucoma^72^, ^73^ can be seen in Figure 2.

#### 2.4.1 Genetic models of RP

The first retina degeneration model discovered as early as 1924 in mice showed mutations in rod phosphodiesterase *Pde6b* gene and later was shown to be involved in RP.^74^ Retinitis pigmentosa is the largest subgroup of inherited retinal diseases and affects more than 1 million people worldwide.^5^ Using this information, many *Pde6* mutated animal models are created to copy the disease.^5^, ^74^, ^75^ For example, in the case of *Pde6b^rd1/rd1^* mouse models, a null mutation creates a loss of rod cells within 2 weeks.^75^ Recent RP models include *Pde6a^V68 M/R562W^* compound heterozygotes in mice, which is an accurate homolog of human RP.^76^ *Pde6b^−/−^*, *Prkdc^−/−^* is another novel immunocompromised RP model developed in mice.^77^ It is produced by a cross between *Pde6b^rd1/rd1^* mutants and *Prkdc^−/−^* mutants that lack the expression of B cells, T cells, and natural killer T (NKT) cells. Thus, these double homozygotes help us understand the disease progression from both a genetic and an immunological viewpoint.^77^

Rhodopsin mislocalization is often seen in RP patients; utilizing this information, *rho_Q344ter_‐dend2 Xenopus laevis* transgenic models have been created in which human mutated rhodopsin is fused with Dendra fluorescent protein, and rhodopsin terminal amino acids are expressed in *Xenopus* to model RP.^78^ CRISPR‐Cas9 edited rhodopsin in *Xenopus* is another recent model of RP.^79^ In zebrafish, morpholino‐based ceramide kinase‐like *cerkl* gene knockdown and expression of an autosomal dominant form of rhodopsin (Q344X) have been used to model RP.^80^, ^81^ Apart from the genes mentioned above, approximately 67 genes have been mapped to be involved in heterogenous RP and thus present targets for animal model development.^75^, ^82^

#### 2.4.2 Genetic models of DR

Diabetic retinopathy is an associated complication of diabetes mellitus and is shown in one‐third of the diseased patients.^63^ There are 5 genetic models of DR in mice; of these, leptin receptor deficient mouse model *Lepr^db^* dates back to the 1990s and shows association with type 2 diabetes.^83^ *Ins2^Akit^*, on the other hand, mimics type‐1 diabetes associated DR and happens as a result of *Insulin 2* gene missense mutation.^63^, ^84^ The amenability of forward and reverse genetics makes zebrafish another valuable model for genetic manipulation. To mimic DR in zebrafish, mutations in the von Hippel‐Lindau tumor suppressor gene are created providing us with *vhl^−/−^* zebrafish^85^, ^86^ that phenotypically mimic detachment of the retina, vascular leakage, and macular edema.^87^ Another novel model for DR is well exemplified by *INS* ^C94Y^ transgenic pig model made via expression of mutation insulin gene.^88^

## 3 ACTIVATION OF RETINAL STEM CELLS

As the name suggests, retinal stem cells (RSC) are progenitor cells specific to the retina and are induced exclusively to form the retinal cells. They are present throughout the vertebrate lineage^3^ but, in comparison with lower vertebrates higher vertebrates such as mammals, are incapable of employing the regenerative property as opposed to their counterparts.^8^ This biased ability to regenerate may be due to the presence of stem cells alone not conferring the capability to regenerate but instead requiring a highly coordinated activity between the stem cell and its niche,^6^ which seems to be lacking in the case of mammals. These so‐called niches are microenvironmental compartments that sustain neural stem cell and endow them the properties of self‐renewal and lineage differentiation.^89^ Certain conditions needed for creation of this proliferative niche include proper mode of stimulation in the form of injury, followed by epigenetic chromatin alterations, expression of specific transcription factors, assembly of growth factors and immune cells, cell signaling initiation, and switching of cells from a glycolytic to the oxidative pathway.^6^, ^7^, ^90^ In the upcoming section, we have provided a list of retinal stem cells found in different vertebrates and briefly discussed the genes, growth factors, and signaling pathways that, together with these stem cells, help create a proper microenvironment for RSC activation among different vertebrate models. These have also been summarized in Table 1.

**TABLE 1:** List of endogenous retinal stem cells source across the vertebrate lineage, their characteristics (in vivo), and an updated list of growth factors, cytokines, and signaling pathways discovered using different animal models involved in retina regeneration

Model organisms	Source of RSC	Characteristics of endogenous RSC (in vivo)		Growth factors and cytokines	Signaling pathways	
					Stimulatory	Inhibitory
Fish (zebrafish)	1.CMZ	Proliferate Differentiate	+ +	HbEgf, Fgf, Insulin, ILGF, Leptin, Midkine, PdGF.^17^, ^18^, ^108^, ^111^, ^112^, ^113^, ^114^	Wnt/β‐catenin, Jak/stat, Hedgehog, Insm1, Tgfβ signaling.^4^, ^17^, ^92^, ^108^, ^114^, ^116^, ^117^, ^118^, ^119^, ^122^, ^123^	Let7 microRNA, Dkk Notch, Insm1, Tgfβ signaling.^17^, ^102^, ^108^, ^116^, ^120^, ^121^, ^122^, ^123^
	2.RPE	Proliferate Differentiate	+ +			
	3.MG	Proliferate Differentiate	+ +			
Amphibians	1.MG	Proliferate Differentiate	+ −	Fgf, Heparin^134^, ^140^, ^143^, ^144^	Wnt/β‐catenin, Hedgehog, MAPK, Heparin‐Thrombin pathway.^132^, ^134^, ^135^, ^140^, ^143^, ^144^	
	2.CMZ	Proliferate Differentiate	+ +			
	3.RPE	Proliferate Differentiate	+ +			
Birds	1.CMZ	Proliferate Differentiate	+ +	FGF, Insulin, HB‐EGF, BMP, retinoic acid, kainic acid.^141^, ^148^, ^149^, ^150^, ^151^, ^152^, ^153^, ^154^, ^155^, ^156^, ^157^, ^163^, ^164^	SHH/FGF/Erk, Canonical BMP, FgFR/MEK/Erk, Jak‐stat, Notch, mTor, hedgehog.^142^, ^148^, ^150^, ^151^, ^153^, ^161^, ^162^	β‐Catenin, Hedgehog, Glucocorticoid signaling.^148^, ^149^, ^159^
	2.IPE*	Proliferate Differentiate	− −			
	3.RPE*	Proliferate Differentiate	− −			
	4.MG	Proliferate Differentiate	+ +			
Mammals	1.CB	Proliferate Differentiate	− −	FGF, Retionic acid, insulin, EGF, HB‐EGF & progranulin.^156^, ^184^, ^185^	Notch signaling, C‐kit, WNT, mTor, hedgehog.^170^, ^171^, ^175^, ^188^, ^189^, ^191^	Hippo pathway, Nf1.^182^, ^186^
	2.RPE	Proliferate Differentiate	+ +			
	3.IPE*	Proliferate Differentiate	− −			
	4.MG*	Proliferate Differentiate	− −			

Note

“+” indicates in vivo proliferation and differentiation; “−” indicates absence of in vivo proliferation and differentiation.

*Studies on bird and mammalian IPE have shown their ability to proliferate but only in in vitro condition as of now.^148^, ^178^ *On the other hand, RPE in birds and MG in mammals require external supplementation of growth factors or manipulated gene expression for successful reprogramming.^153^, ^182^, ^183^

## 4 RETINAL STEM CELLS IN DIFFERENT VERTEBRATES

### 4.1 Retinal stem cells in teleost fishes

The most remarkable ability to regenerate the retina among the vertebrates is displayed by teleost fishes such as the zebrafish,^91^ where the growth of retina takes place throughout their life.^3^ The regions that have been identified in zebrafish with retinal stem cells include the ciliary marginal zone (CMZ), retinal pigment epithelium (RPE), and the Müller glia (MG).^92^, ^93^

#### 4.1.1 CMZ in zebrafish

Lying between the “*mitf*” gene expressing RPE and “*vsx2*” gene expressing neural retina, the CMZ is a zone of persistent neurogenesis^25^ and can express retinal progenitor associated eye field transcription factors (EFTFs)^89^, ^94^ as well as proneural genes such as *ngn2* and *ascl1*.^94^ They can generate retinal cell types such as photoreceptors (cones), inner retinal neurons, and RPE upon injury.^95^

#### 4.1.2 RPE in zebrafish

Retinal pigment epithelium, which lies between the neural retina and choroid, performs a host of functions and happens to be a prominent source of RSC in lower vertebrates.^93^ Investigations have shown cellular repair to occur via transdifferentiation in the case of RPE‐mediated regeneration.^96^ Yet, relatively little is understood about the biology of RPE‐mediated regeneration in higher vertebrates. Recent studies involving zebrafish have presented some new insights into this topic. The transgenic zebrafish line *rpe65a:nfsB‐eGFP* enables retinal pigment epithelial cell specific expression of nitroreductase (nfsB) and green fluorescent protein (GFP) under the control of *rpe65a* enhancer, which facilitates NTR‐Mtz‐mediated RPE ablation.^93^, ^97^ This study has shown RPE to follow a Wnt‐mediated reparative pathway^93^ and has accounted for the presence of macrophages and microglia indispensable for timely progression of proliferation.^98^

#### 4.1.3 Müller glia in zebrafish

The molecular characterization of neuronal stem cells of the brain showed expression of properties identical to radial glia cells,^99^, ^100^ therefore indicating that radial glial cells are the progenitors of neurons. Since the retina is also derived from the CNS and possesses its own glial cells, the “Müller glia,” these cells were a potential target for identifying stem cell progenitors in the eye. MG in zebrafish is a potent source of neuronal progenitor and post‐injury become reactivated via expression of certain pluripotency genes such as *pax6b*,^101^, ^102^ *ascl1*,^17^, ^33^, ^103^ *lin28*,^17^, ^95^ and *stat3*.^17^, ^104^ Ongoing research has been able to add more to this list. Gene expression analysis of *sox2* morpholino knockouts has also shown to cause a significant reduction in *ascl1a* and *lin28* levels, which states its importance in reprogramming.^105^, ^106^ Myc is another well‐known pluripotency inducing transcriptional activator,^107^ and in zebrafish it has been found to regulate *lin28* positively via *ascl1a* expression and negatively via Myc histone deacetylase (*hdac*) inhibition of *her4.1*, a delta notch effector.^108^ Oct4 is another transcription factor that, apart from upregulating regeneration‐associated genes, regulates proliferation and cell cycle exit respectively via inhibiting and eventually inducing transforming growth factor β (Tgfβ) signaling.^18^

Several cytokines and growth factors are produced post‐injury from MG, and some of these are known to cause diseases such as proliferative vitreoretinopathy in humans.^109^, ^110^ However, in regeneration‐competent organisms, where timely termination of proliferation occurs, some cytokines and growth factors have been shown to positively regulate regeneration. In the case of zebrafish, these include heparin‐binding epidermal growth factor (HbEgf), fibroblast growth factor (Fgf), insulin, insulin‐like growth factor (Igf), leptin, etc^111^, ^112^, ^113^, ^114^, ^115^ Tumor necrosis factor alpha (Tnfα) is another cytokine that regulates regeneration as it works upstream of Ascl1a.^114^ Midkine is another addition to the list and facilitates G1‐to‐S transition.^116^ Furthermore, blocking growth factors such as Tgfβ^18^ and platelet‐derived growth factor^19^ also tend to downregulate regeneration‐associated transcription factors and are essential for regeneration.

The major signaling pathways already known to be involved in retina regeneration that positively regulates regeneration include Wnt/β catenin pathway,^111^, ^115^, ^117^, ^118^ Jak‐stat pathway,^119^ and Pik3/Akt signaling pathway.^4^ Hedgehog pathway (Shh) is another known pathway involved in retina regeneration and can control both the proliferation and differentiation of MG.^120^ Signaling pathways that inhibit regeneration include the Notch pathway,^111^, ^121^, ^122^ let‐7 microRNA,^103^ and Dikkopf (Dkk) pathway.^117^ Certain pathways seem to play both proproliferative and antiproliferative roles to drive differentiation depending upon their temporal expression. Insm1a‐dependent pathway is one such pathway and can inhibit *ascl1a* expression and induce *ascl1a*‐mediated *dkk* gene repression.^123^, ^124^

Furthermore, it can regulate controlled proliferation by inhibiting *hb‐egfa* gene expression.^116^ Tgfβ‐mediated upregulation of matrix metalloproteinase *mmp2* during the earlier phases of injury promotes MG proliferation, and its recombinant form upregulates regeneration‐associated genes viz. *oct4*, *ascl1a*, *lin28*, etc^18^ Later stages show Tgfβ‐mediated proliferative gene repression via activation of the neuroD complex for cell cycle exit.^18^

### 4.2 Retinal stem cells in amphibians

Amphibians, like the teleost fishes, possess massive regeneration potential. Urodele newt, salamander, and anuran *Xenopus* are popular models for eye regeneration studies.^125^, ^126^, ^127^ Retinal stem cells in amphibians have also been identified in the MG, CMZ, and RPE.

#### 4.2.1 Müller cells in amphibians

Until very recently, MG in the case of amphibians was considered not to possess any proliferative abilities. However, detection of proliferating cells in newts and *Xenopus* larvae^128^, ^129^ has paved the way for MG to be a probable source.^128^, ^129^ While current findings support this idea, a recent experiment has revealed more profound reactivation in cells from older organisms than from younger ones,^130^ which opposes the general feature of regeneration; hence, MG cells in amphibians need further investigation.

#### 4.2.2 CMZ in amphibians

Similar to the fishes, amphibians such as *Xenopus* also express RSC markers along the peripheral region of CMZ and follow a centripetal pathway of retinogenesis during development and regeneration.^131^, ^132^, ^133^, ^134^ Stem cell markers such as *pax6* and *six3* show high expression at the periphery; this is followed by neurogenic genes such as *delta* and *notch*, and finally proneural genes such as *neuroD* towards the center.^131^

In the past decade, protooncogenes such as *c‐myc* and *n‐myc*, which happen to be important cell cycle regulators, have also been shown to have centripetally increasing expression.^132^ Signal transduction pathways also work simultaneously for successful development and regeneration and, to name a few, include the canonical Wnt and the Shh pathway.^133^, ^135^, ^136^ While the former promotes proliferation, the latter promotes both proliferation and differentiation via Sprf1‐mediated Wnt inhibition.^133^

#### 4.2.3 Retinal pigment epithelium in amphibians

The other important mode of retina regeneration in amphibians is “transdifferentiation” of the RPE seen in urodeles^137^, ^138^ and more recently has also been shown in anurans.^126^ This is a mechanism where the cell dedifferentiates and returns to a point where it can switch lineages and hence redifferentiate into another cell type.^139^

Investigating the molecular mechanism underlying RPE‐mediated retinogenesis has shown that RPE goes into a brief, unique multipotent state, and unlike mammals where RPE proliferation is associated with PVR, amphibian RPE is well reprogrammed for recovery.^140^, ^141^, ^142^ This reprogramming involves the expression of pluripotency factors such as C‐myc, Klf4, and Sox2 post injury which takes cells to a dedifferentiated state.^143^ This follows the expression of *pax6* and *mift* genes that accounts for redifferentiation. These RPE cells then undergo cell cycle entry initiated by Fgfs and Igf1, which in turn induce MEK‐Erk and heparin‐susceptible pathways.^141^ In the case of newts, apart from the expression of EFTFs of which *pax6* shows 2 variants,^143^ multipotency state regulates via expression of pluripotency factors C‐myc, Klf4, and Sox2.^140^ In the case of *Xenopus*, RPE‐mediated regeneration depends on Fgf‐dependent MAPK pathway.^144^, ^145^

### 4.3 Retinal stem cells in birds

In the case of birds, the chick is used as a popular model organism for studying retina regeneration. The CMZ, iris pigment epithelium (IPE), RPE, and MG are 4 regions that have been identified to contain retinal stem cells in the chick.^142^, ^146^, ^147^, ^148^

#### 4.3.1 CMZ in chick

The expression of multipotent progenitor associated transcription factors and incorporation of BrdU at the peripheral margins of retina in chick have shown the capability of cells in CMZ to undergo regeneration.^137^ However, unlike their cold‐blooded counterparts, CMZ in chick possesses restricted capabilities and can only produce bipolar and amacrine cells.^137^ Signaling pathways such as sonic hedgehog alongside FGF signaling^149^ have been shown to facilitate cellular reprogramming in chick CMZ. Moreover, both pathways are interdependent and work via Erk pathway.^150^ Beta‐catenin pathway in chick CMZ, on the contrary, hinders entry into the cell cycle.^151^ The canonical BMP pathway is another important regulator of regeneration that induces regeneration via activation of Smad and upregulation of FGF signaling.^152^

#### 4.3.2 Iris pigment epithelium in chick

Cell culture studies on IPE derived from postnatal chickens have shown multipotent progenitor expression and, thus, the capability to proliferate and undergo depigmentation. Hence, they are also as a potential source of RSC.^148^

#### 4.3.3 Retinal pigment epithelium in chick

Similar to amphibians, RPE‐mediated transdifferentiation for retina regeneration is also known to occur in birds. Postinjury transcription factors along with pluripotency inducing factors viz. SOX2, CMYC, and KLF4 are expressed transiently.^153^ RPE in chick has also shown FGF to be an essential player in regeneration,^147^, ^154^ and signaling occurs via fibroblast growth factor receptor (FGFR)/MEK/Erk pathway^155^ with *Lin28* as one of the downstream targets.^147^ Contrary to the CMZ, the SHH pathway in RPE has been shown to inhibit the transdifferentiation process.^149^

#### 4.3.4 Müller glia in chick

MG is the most studied retinal stem cell niche in the case of the chick, owing to its expression of proliferation markers such as PAX6, ASCL1, CEH10, NOTCH, FOXN4, etc,^138^, ^156^ which renders it high neurogenic potential. Like RPE and CMZ in chick MG, FGF is a key player that contributes well to proliferation.^150^, ^157^ The use of insulin,^157^ HB‐EGF,^158^ and bone morphogenetic protein (BMP)^159^ has also shown positive results. Notch signaling,^149^, ^160^ glucocorticoid receptor signaling,^161^ and sonic hedgehog signaling^162^ are some known pathways that regulate MG proliferation in birds. Recent additions to the list include the following: Jak/stat pathway, which promotes MG progenitor cell proliferation but has been shown to be not as efficient in neuronal differentiation^163^; another mammalian target of rapamycin (mTOR) pathway has also been shown to promote MG reprogramming as its inhibition has a significant effect on PAX6 levels. Stimulatory pathways such as hedgehog and WNT are also blocked on mTOR inhibition.^164^ Activation of retinoic acid signaling pathway^165^ and growth hormone treatment^166^ have also surfaced as recent findings in chick MG regeneration.^144^, ^145^ Moreover, the existence of crosstalk between FGF and notch^139^ and FGF and hedgehog,^141^ and its capability to activate the Jak‐stat pathway^142^ and mTOR signaling,^143^ makes it a focal point in the case of chick regeneration.

### 4.4 Retinal stem cells in mammals

The regenerative potential of mammalian retina has long been questioned owing to their incapability to “self‐heal” upon injury or pathological conditions. However, this “incapability” has proved to be a myth owing to the success of experiments that have shown the presence of RSC existing in the quiescent form in mammals in different locations within the retina. These include the ciliary body (CB), IPE, RPE, and MG.^127^ The knowledge gathered from regeneration‐competent animal models and its application in mammals has helped unravel certain intrinsic and extrinsic factors and pathways that induce retinal stem cell activation.

#### 4.4.1 The ciliary body in mammals

The CB or retinal margin in higher vertebrates represents a location of stem cells that corresponds to the ciliary marginal zone in the case of lower vertebrates.^167^, ^168^, ^169^ In vitro analysis of the CB in both mice and humans shows that they are capable of forming neurospheres that express RSC markers and generate most retinal cell types, including photoreceptors.^170^, ^171^ Notch, WNT, and C‐kit are the signaling pathways that can regulate CB stem cell activation.^172^, ^173^

#### 4.4.2 Retinal pigment epithelium and iris pigment epithelium in mammals

Subpopulations of cells both in the RPE and the IPE have shown retinal progenitor properties^155^ making them a probable target for studying eye regeneration.^143^, ^174^ In the case of mammalian RPE, peripheral portions of rat retina have shown the ability to enter the cell cycle and proliferate. Similarly, experiments with dedifferentiated RPE transplanted into injured rat eye and human eye have also shown successful repairment.^175^, ^176^ A recent finding suggests the involvement of mTOR signaling for RPE‐mediated regeneration in humans.^177^

In the case of IPE cells, in vitro studies on rodent and pigs have also demonstrated the ability of IPE to proliferate on addition of fibroblast growth factors.^178^

#### 4.4.3 Müller glia in mammals

Unlike fish and amphibian MG, the MG in mammals tends to maintain its quiescence even postembryonically and respond to injury with prolonged reactive gliosis.^179^, ^180^ That mammalian MG also possessed the capability to evade this quiescence was first confirmed in a retinal explant culture study of retina taken from NMDA‐injured Sprague Dawley rats, which managed to produce bipolar cell rod photoreceptors.^53^ *Ascl1a* overexpression alongside a histone deacetylase inhibitor has been shown to relatively upregulate MG proliferation in mice; however, the effects seem to be transient.^181^, ^182^ Findings in a recent paper have shown that the transient nature of proliferation is due to the presence of Nuclear factor 1 (Nf1), a transcription network that inhibits *Ascl1a* and works to promote reactive gliosis instead of regeneration in mammals.^183^ Another gene, *Lin28a*, a posttranscriptional regulator, can also induce reprogramming as it can control the formation of MG or other neural lineages depending upon its expression or deletion.^184^, ^185^ Neurogenin‐2, a basic helix‐loop‐helix (bHLH) transcription factor, is another potential candidate that activates MG regeneration.^186^ Taking hints from regenerative models, many growth factors have been identified that help stimulate MG differentiation in mammals. These include retinoic acid, FGF, insulin, epidermal growth factor (EGF), HB‐EGF, and progranulin.^158^, ^187^, ^188^ Regarding the signaling pathways involved, the most recent inclusion is the “hippo pathway,” which is a conserved kinase pathway that works during development.^189^ Hippo pathway‐mediated phosphorylation of transcriptional cofactor Yes‐associated protein (YAP) influences cyclin D1 levels required for the initial burst in MG reprogramming.^190^ Apart from these well‐known signaling pathways that are already known to us, the WNT and Notch pathway positively regulates MG proliferation,^191^, ^192^ with Notch acting as an inhibitor of CDK inhibitor p27^kip^.^1193^ Intraocular injections of SHH also stimulate MG proliferation and enhance neurogenic potential.^194^

## 5 DISCUSSION

In vertebrates, the “regenerative neurogenesis,” that is, the ability of postembryonic establishment of functional neuron regeneration, is said to exist,^195^ but this feature appears to be compromised in higher vertebrates. Analysis of the above‐mentioned endogenous retinal stem cells have helped to unveil species‐specific trends and pathways followed during retina regeneration, revealing what appears to be missing in mammals. For instance, while the CMZ shows high regenerative potential in fish and amphibians, chick and mammalian CMZ seems to require the presence of additional mitogens and signaling factors.^196^ Similarly, in comparison with the amphibian RPE that transdifferentiates its way to regenerate the retina, mammalian RPE seems to have preserved only a certain fraction of the ability to proliferate.^174^ Moreover, the regulatory elements required for induction of transdifferentiation seem to be lacking in mammals.^174^, ^196^ Regarding the Müller glia, *Ascl1* upregulation appears to be key in facilitating retina regeneration both in fish and birds. MG‐specific expression of *Ascl1* in combination with a histone deacetylase inhibitor has shown to activate regenerative potential in mammals as well, but the results appear to be transient.^182^ Recent experiments have shown that this state of dormancy of mammalian MG is mediated by a dedicated gene regulatory network that upregulates upon injury.^183^

The evolutionary aspect of regeneration is far from being understood. However, loss of such recuperative powers in higher vertebrates has been speculated to occur for resolving wounds in the best possible way and to increase the reproductive fitness.^197^ In reality, loss of vision in humans has the potential of exacerbating tremendous socioeconomic pressure. Animal models of retina regeneration present us with an opportunity to solve this mystery utilizing an “in vivo” platform that allows for understanding complex interactions happening during the process. These models have allowed the establishment of injury mechanisms that happen to be the first‐hand stimulus for RSC activation and hence allow us to explore the underlying molecular mechanisms. Furthermore, they also serve excellently as eye disease models, thereby helping to understand the pathophysiology of the disease and, hence, designing and trial of therapeutic drugs. The findings in one animal model pave way for experimenting with the same in another animal model and allow for a broader understanding of relatedness or heterogeneity of retina regeneration activation among different species. The information thus gathered from these models about the underlying regeneration‐associated molecules and pathways can ultimately be used as experimental targets in mammalian models and gradually in humans.

## CONFLICT OF INTEREST

The authors state no conflicts of interests.
